# Safety and feasibility of 2 h of normothermic machine perfusion of donor kidneys in the Eurotransplant Senior Program

**DOI:** 10.1093/bjsopen/zraa024

**Published:** 2021-01-09

**Authors:** E Rijkse, J de Jonge, H J A N Kimenai, M J Hoogduijn, R W F de Bruin, M W F van den Hoogen, J N M IJzermans, R C Minnee

**Affiliations:** Division of Hepato-Pancreato-Biliary and Transplant Surgery, Department of Surgery, Erasmus MC University Medical Centre, Rotterdam, the Netherlands; Division of Hepato-Pancreato-Biliary and Transplant Surgery, Department of Surgery, Erasmus MC University Medical Centre, Rotterdam, the Netherlands; Division of Hepato-Pancreato-Biliary and Transplant Surgery, Department of Surgery, Erasmus MC University Medical Centre, Rotterdam, the Netherlands; Nephrology and Transplantation, Department of Internal Medicine, Erasmus MC University Medical Centre, Rotterdam, the Netherlands; Division of Hepato-Pancreato-Biliary and Transplant Surgery, Department of Surgery, Erasmus MC University Medical Centre, Rotterdam, the Netherlands; Nephrology and Transplantation, Department of Internal Medicine, Erasmus MC University Medical Centre, Rotterdam, the Netherlands; Division of Hepato-Pancreato-Biliary and Transplant Surgery, Department of Surgery, Erasmus MC University Medical Centre, Rotterdam, the Netherlands; Division of Hepato-Pancreato-Biliary and Transplant Surgery, Department of Surgery, Erasmus MC University Medical Centre, Rotterdam, the Netherlands

## Abstract

**Background:**

The 5-year graft survival rate of donor kidneys transplanted in the Eurotransplant Senior Program (ESP) is only 47 per cent. Normothermic machine perfusion (NMP) may be a new preservation technique that improves graft outcome. This pilot study aimed to assess safety and feasibility of this technique within the ESP.

**Methods:**

Recipients were eligible for inclusion if they received a donor kidney within the ESP. Donor kidneys underwent 2 h of oxygenated NMP with a red cell-based solution at 37°C, additional to standard-of-care preservation (non-oxygenated hypothermic machine perfusion). The primary outcome was the safety and feasibility of NMP. As a secondary outcome, graft outcome was investigated and compared with that in a historical group of patients in the ESP and the contralateral kidneys.

**Results:**

Eleven patients were included in the NMP group; the function of eight kidneys could be compared with that of the contralateral kidney. Fifty-three patients in the ESP, transplanted consecutively between 2016 and 2018, were included as controls. No adverse events were noted, especially no arterial thrombosis or primary non-function of the transplants. After 120 min of oxygenated NMP, median flow increased from 117 (i.q.r. 80–126) to 215 (170–276) ml/min (*P* = 0.001). The incidence of immediate function was 64 per cent in the NMP group and 40 per cent in historical controls (*P* = 0.144). A significant difference in graft outcome was not observed.

**Discussion:**

This pilot study showed NMP to be safe and feasible in kidneys transplanted in the ESP. A well powered study is warranted to confirm these results and investigate the potential advantages of NMP on graft outcome.

## Teaser

This pilot study aimed to assess the safety and feasibility of normothermic machine perfusion (NMP) for donor kidneys transplanted in the Eurotransplant Senior Program. NMP is a safe technique and may improve the incidence of immediate function. A well powered study is warranted to confirm these results and to investigate the potential advantages of NMP on graft outcome.

Potential to improve graft outcome

## Resumen

**Antecedentes:**

La supervivencia a cinco años del injerto renal en el *Eurotransplant Senior Program* (ESP) es únicamente del 47%. La perfusión normotérmica mecánica (*normothermic machine perfusion*, NMP) puede ser una nueva técnica de preservación para mejorar el resultado del injerto. Este estudio piloto tuvo como objetivo evaluar la seguridad y la viabilidad de esta técnica dentro del programa ESP.

**Métodos:**

Se incluyeron los receptores de un riñón dentro del ESP. Los riñones de los donantes se sometieron, además de la preservación estándar (perfusión hipotérmica no oxigenada), a 2 horas de NMP oxigenada con una solución a base de glóbulos rojos a 37°C. La variable principal fue la seguridad y la viabilidad de la NMP. Como variable secundaria, se analizó el resultado del injerto y se comparó con un grupo histórico de pacientes del ESP y con los riñones contralaterales.

**Resultados:**

En el grupo NMP se incluyeron 11 pacientes; de los que en 8 riñones se pudo comparar su función con el riñón contralateral. Se incluyeron como controles 53 pacientes del ESP, trasplantados consecutivamente entre el 2016 y el 2018. No se observaron efectos adversos, remarcando la ausencia de trombosis arterial o disfunción primaria del injerto. Tras los 120 minutos de NMP oxigenada, la mediana de flujo aumentó de 117 ml/min (rango intercuartílico, IQR 80-126) a 215 ml/min (IQR 170-276) (*P* = 0,001). La incidencia de función inmediata fue del 64% en el grupo de NMP y del 40% en los controles históricos (*P* = 0,144). No se observó diferencia significativa en el resultado del injerto.

**Conclusión:**

Este estudio piloto mostró que la NMP es segura y factible en los riñones trasplantados dentro del ESP. Se justifica la realización de un estudio con suficiente poder estadístico para confirmar estos resultados e investigar las posibles ventajas de la NMP en el resultado del injerto.

## Introduction

Kidney transplantation is the only life-saving treatment for end-stage renal disease^1^. Because the demand for suitable donor kidneys exceeds the supply, death while being waitlisted for a donor kidney to become available is a major problem^2^. One solution to increase the donor pool is the more frequent use of expanded criteria donor (ECD) and donation after circulatory death (DCD) kidneys. Unfortunately, ECD kidneys are known to have a 70 per cent increased risk of graft failure, resulting in earlier return to dialysis or retransplantation^3^. One example of the use of these ECD and DCD kidneys is the Eurotransplant Senior Program (ESP), in which kidneys from donors aged 65 years or more are allocated to recipients aged 65 years or above, focusing on short ischaemia time to minimize additional organ damage^4^. Unfortunately, ESP results have shown high rates of delayed graft function (DGF) and primary non-function (PNF), with a 5-year graft survival rate of only 47 per cent^5^. Especially for DCD kidneys transplanted within the ESP, the reported incidence of DGF and PNF is 74.1 and 12.4 per cent respectively^6^. Therefore, attempts to improve outcomes in this particular group of recipients are important.

In the past decade, machine perfusion has regained interest as a promising preservation technique for deceased donor organs. Moers and colleagues^7^ showed that preservation with hypothermic machine perfusion (HMP) improved clinical outcomes after kidney transplantation in comparison with static cold storage (SCS). Unfortunately, in this RCT, patients transplanted in the ESP had minimal benefit from HMP^8^. Additional preservation methods may be needed in these suboptimal donor kidneys, such as normothermic machine perfusion (NMP). NMP has several advantages in addition to HMP alone. First, NMP allows for pretransplantation viability assessment as the kidney becomes metabolically active. Donor kidney function can be assessed before transplantation, which has been shown to reduce discard rates^9^. Second, in contrast to HMP, NMP can improve kidney function through active repair and reconditioning. NMP itself may improve kidney function as previous clinical studies showed a decreased incidence of DGF compared with that in donor kidneys not receiving NMP^10^^,^^11^. In addition, NMP can provide a platform for kidney-tailored organ repair by adding therapies ‘on the pump’, such as mesenchymal stem cells^12^^,^^13^. This may be of special interest in suboptimal donor kidneys, such as those transplanted within the ESP population. Third, NMP could extend preservation time without prolonging ischaemia time. This could be beneficial for several reasons, such as more time to find an appropriate match and preparation of the operative procedure. When there are several kidney offers at the same time, NMP can delay transplantation safely without compromising the quality of the graft because of prolonged cold ischaemia time.

Although NMP is theoretically attractive, its application in deceased donor kidney transplantation is largely unknown, as only a few centres have published on this technique in a clinical kidney transplantation setting^10^^,^^11^^,^^14^^,^^15^. NMP is more complex, expensive, and sensitive to technical problems such as inhomogeneous perfusion, perfusate toxicity, and perfusion-related organ damage. Moreover, in contrast to HMP, failure of NMP leads to detrimental extra warm ischaemia time. Therefore, the aim of this study was to investigate the safety and feasibility of implementing 2 h of NMP in the ESP.

## Methods

This prospective, single-centre pilot study aimed to include 10 ESP recipients of a technically successful NMP procedure. Between 1 January 2018 and 1 December 2018, all waitlisted patients aged 65 years or above were asked to participate. Patients were excluded if they received a dual kidney transplant. Multiple renal arteries in the donor organ were not a contraindication for inclusion as arterial reconstruction could be performed safely before the NMP procedure. All kidneys were allocated according to the regular Eurotransplant rules based on blood-type compatibility and waiting time. All donor kidneys included in the study were suitable for transplantation based on standard criteria before the start of NMP. Both donation after brain death (DBD) and DCD kidneys were included. The study was performed in Erasmus Medical Centre, Rotterdam, the Netherlands, and approved by the local medical ethics committee (POSEIDON study, MEC 2017-503). All recipients provided informed consent to participate in the study.

### Kidney procurement

The donor procedure was carried out in the donor hospital, according to the standard Eurotransplant retrieval procedure. For DCD donors, a 5-min period of ‘no touch’ after asystole was maintained, according to Dutch law. Thereafter, the donor was transported immediately to the operating room. The super-rapid recovery technique was used to perform aortic flush with at least 5 litres of ice-cold (0–4°C) Belzer University of Wisconsin (UW^®^) Cold Storage Solution (Bridge to Life, London, UK) to which 25 000 or 50 000 units heparin were added, depending on kidney-only or multiple organ procurement. After procurement, the donor kidney was stored and transported on pulsatile, non-oxygenated HMP using the LifePort^®^Kidney Transporter (Organ Recovery Systems, Itasca, IL, USA) or Kidney Assist Transport^®^ (Organ Assist, Groningen, the Netherlands), as this is standard of care in the Netherlands. In other Eurotransplant countries, kidneys are still preserved on SCS.

### Normothermic machine perfusion and transplantation procedure

Upon arrival of the donor kidney at Erasmus Medical Centre, the kidney was disconnected from the HMP device under sterile conditions. Thereafter, acceptability for transplantation was assessed. The Kidney Assist (Organ Assist, Groningen, the Netherlands) used for NMP provides pressure-controlled perfusion with a rotary pump. Perfusion was performed with an oxygenated, plasma-free, red cell-based solution, based largely on the perfusate, as described by Hosgood *et* al.^14^. The composition of the perfusate is detailed in *Table 1*. After preparation of the Kidney Assist, the renal artery was cannulated with a sterile 3-mm PVC funnel (Teleflex, Athlone, Ireland). The kidney was then connected to the Kidney Assist.

**Table 1:** Composition of perfusate used for normothermic machine perfusion

Content	Final concentration
**Compatible cross-matched blood (ml)**	250
Sterofundin^®^ solution (ml)*	500–1000
**Mannitol 15% (ml)**	10–20
**Dexamethasone 4 mg (ml)**	2
**Sodium bicarbonate 8.4% (ml)**	10–40
**Heparin 1000 units/ml (ml)**	2–4
**Augmentin 1.2 g (ml)**	10
**Prostacyclin 0.5 mg (ml/h)**	4
**Glucose 5% (ml/h)**	7
Nutriflex^®^ infusion* with the following added	20
** **Insulin (units)	100
** **Sodium bicarbonate 8.4% (ml)	25
** **Multivitamins (ml)	5

*B. Braun, Melsungen, Germany.

Oxygenated NMP was started at 37°C with 100 per cent oxygen at a pressure of 60 mmHg, which was increased manually if deemed necessary based on the colour of the kidney. Urine produced during NMP was recirculated to keep the volume constant, as this has been shown to maintain a more physiological perfusate composition and improved flow during NMP^16^. Perfusion parameters during NMP such as flow, intrarenal resistance, and pressure were registered continuously. After 2 h of NMP, the donor kidney was stored on SCS as briefly as possible until the operating team was ready to perform the kidney transplantation. Upon transplantation, the donor kidney was routinely implanted in the iliac fossa and connected to the external iliac artery and vein. The anastomosis between donor ureter and recipient bladder was an extravesical anastomosis with the use of an external splint. Immunosuppression of the recipients consisted of triple therapy with prednisolone, tacrolimus, mycophenolate mofetil and basiliximab as induction therapy. In the case of multiple arteries and renal artery reconstruction, patients received 12 000 units heparin per 24 h for 5 days, as standard of care in the kidney transplant programme.

### Primary and secondary outcomes

The primary outcome of the study was the safety and feasibility of NMP. Any adverse events in the postoperative period or problems during NMP were recorded. Special attention was given to technical issues such as machine failure and inability to perform NMP or decannulation. Unfavourable short-term transplant outcomes, such as PNF, early rejection, graft infection and graft thrombosis, were also noted. Logistical issues were noted, paying particular attention to the duration of cold ischaemia time. The NMP procedure should not prolong cold ischaemia time, as this impacts graft outcome.

Secondary outcomes were graft outcomes, defined as the incidence of immediate kidney function (no DGF/PNF), biopsy-proven acute rejection (BPAR) within 3 months, post-transplantation estimated glomerular filtration rate (eGFR), and graft and patient survival. eGFR was calculated using the Chronic Kidney Disease Epidemiology Collaboration (CKD-EPI) formula. For graft survival, both death-censored and uncensored graft survival were investigated. DGF was defined as the need for dialysis treatment in the first week after transplantation. PNF was defined as a never functioning graft and was diagnosed retrospectively 3 months after transplantation. Graft outcome was investigated and compared with that in two control groups. The first control group was a historical cohort of consecutive patients in the ESP transplanted without the use of NMP between 1 January 2016 and 31 December 2018. In this control group, cold preservation of the kidney was usually performed with HMP, and for incidental cases with SCS. To reduce bias by confounding, a second control group was formed consisting of the contralateral kidney of each included donor kidney, if that kidney was also transplanted.

### Statistical analysis

For baseline characteristics, continuous variables were presented as median (i.q.r.) values, and comparisons were done using the Mann–Whitney *U* test. Categorical variables were presented as numbers and percentages, and compared with the χ^2^ or Fisher’s exact test, based on expected and observed counts. For comparisons made with the contralateral kidney and for correlated measurements, the Wilcoxon signed rank test was used for continuous data and the McNemar test for categorical data. For the outcome eGFR, if graft failure had taken place, an eGFR of 10 ml/min was imputed. If a patient died with a functioning graft, the last observation of eGFR was carried forward. Survival analysis was performed using the Kaplan-Meier method and curves were compared with the log-rank test. Two-sided *P* <0.050 was considered statistically significant. All analyses were performed using GraphPad Prism^®^ version 5.0 (GraphPad, San Diego, CA, USA) and IBM SPSS^®^ Statistics version 25 (IBM, Armonk, NY, USA).

## Results

Between 1 March 2018 and 1 December 2018, 11 deceased donor kidneys transplanted into recipients participating in the ESP received 2 h of NMP. One protocol violation took place, as one of the donors was aged 63 years and 10 months; this became apparent only once the NMP procedure had started.

Baseline characteristics are shown in *Table 2*. Seven of the 11 kidneys were retrieved from a DCD donor. The median age of donors and recipients was 71 (i.q.r. 66–72) and 71 (67–74) years respectively. The majority of patients (8 of 11) had received dialysis before transplantation, with a median time on dialysis of 22.5 (i.q.r. 14.3–45.3) months. Median total cold ischaemia time was 593 (i.q.r. 497–746) min, which consisted of cold ischaemia time until NMP plus a second short cold ischaemia time after NMP until transplantation (median 83 (34–167) min).

**Table 2:** Baseline characteristics of patients receiving a normothermic machine perfused kidney and historical controls

	NMP group (*n*=11)	Historical control group (*n*=53)	*P* ^†^
**Recipient characteristics**			
** **Age (years)^*^	71 (67–74)	70 (69–75)	0.580^‡^
** **Male sex	9 (82)	28 (53)	0.076
** **BMI (mg/kg^2^)^*^	26.7 (24.7–31.0)	27.4 (24.9–30.8)	0.873^‡^
** **Diabetes	2 (18)	22 (42)	0.146
** **Dialysis treatment			0.280
** **None	3 (27)	6 (11)	
** **Haemodialysis	5 (45)	36 (68)	
** **Peritoneal dialysis	3 (27)	11 (21)	
Time on dialysis (months)^*^	22.5 (14.3–45.3)	22.1 (14.4–32.5)	0.699^‡^
HLA mismatch^*^	4 (3–5)	5 (4–5)	0.229^‡^
** **Current PRA^*^	0 (0–4)	0 (0–0)	0.207^‡^
Smoking			0.772
** **Never	4 (36)	24 (45)	
** **Current	2 (18)	6 (11)	
** **Previous	5 (45)	23 (43)	
** **Ischaemic heart disease	2 (18)	12 (23)	0.745
**Donor characteristics**			
** **DBD donor	4 (36)	13 (25)	0.419
** **Age (years)^*^	71 (66–72)	69 (68–73)	0.872^‡^
** **Male sex	7 (64)	27 (51)	0.443
** **BMI (kg/m^2^)^*^	22.2 (18.1–26.1)	25.6 (23.6–28.2)	0.043^‡^
** **First WIT (min)^*^	13 (0–17)	13 (2–15)	0.921^‡^
**Transplant characteristics**			
** **Second CIT (min)^*^	83 (34–167)	–	–
** **Total CIT (min)^*^	593 (497–746)	600 (480–720)	0.893^‡^
** **Second WIT (min)^*^	18.0 (17.0–21.0)	22.0 (17.5–28.5)	0.095^‡^
** **Arterial reconstruction	5 (45)	13 (25)	0.160
** **Cold preservation method			0.426
** **HMP	8 (73)	44 (83)	
** **Static cold storage	3 (27)	9 (17)	

Values in parentheses are percentages unless indicated otherwise:

*values are median (i.q.r.). NMP, normothermic machine perfusion; HLA, human leucocyte antigen; PRA, panel-reactive antibodies; DBD, donation after brain death; WIT, warm ischaemia time; CIT, cold ischaemia time; HMP, hypothermic machine perfusion.

^†^χ^2^ or Fisher’s exact test, except

^‡^Mann–Whitney *U* test.

Five donor kidneys had multiple renal arteries necessitating arterial reconstruction before transplantation. No technical problems occurred during NMP; in particular, no complications due to decannulation, arterial flow or machine failure were noted. Despite the high incidence of vascular multiplicity, no arterial thrombosis occurred during NMP or after transplantation. There were no cases of PNF, and all kidneys were demonstrated to be functioning.

**Fig. 1:**
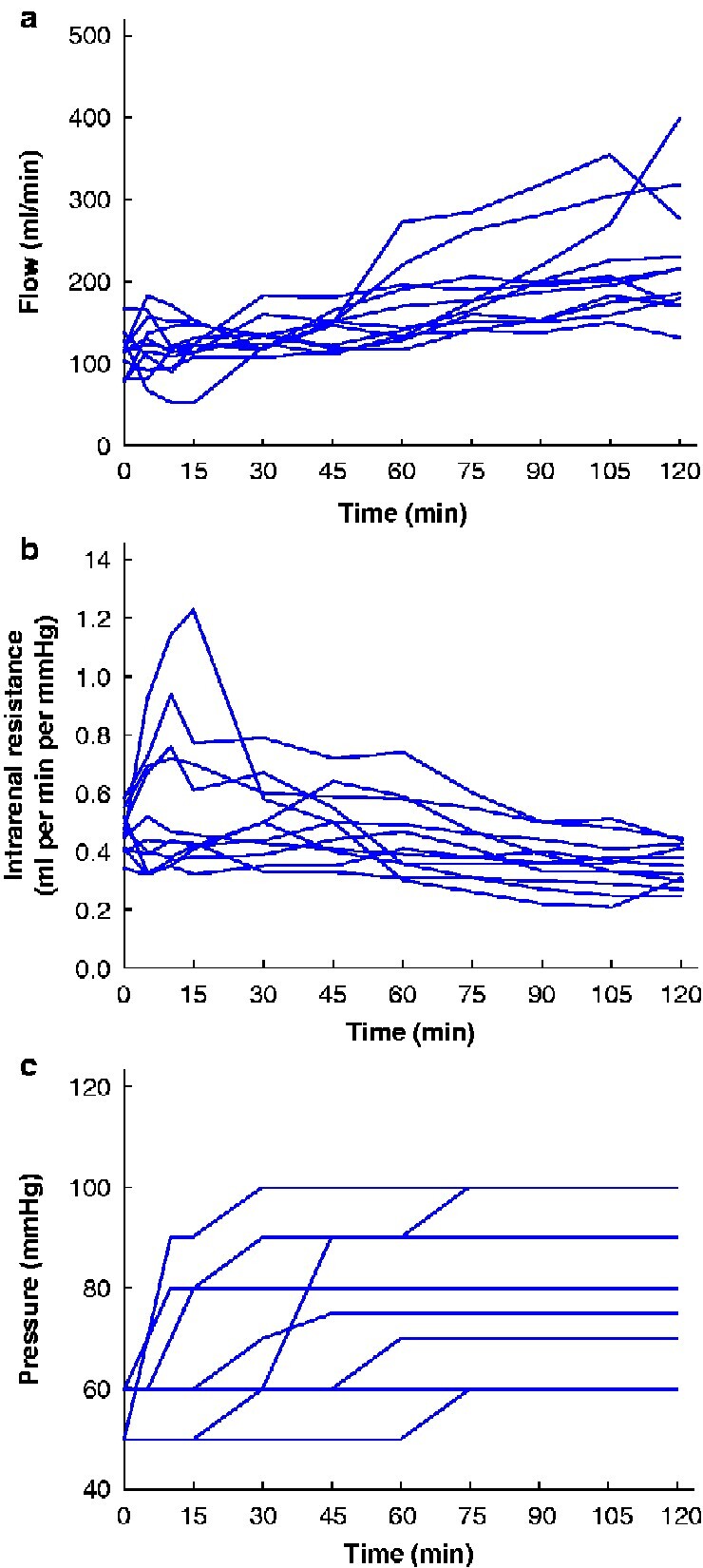
Flow, intrarenal resistance and pressure during normothermic machine perfusion **a** Flow, **b** intrarenal resistance and **c** pressure of the 11 donor kidneys.

*Fig. 1* shows median flow, pressure, and intrarenal resistance during NMP for the 11 recipients, and *Table 3* shows differences between perfusion parameters at the start and end of NMP. Median flow was 117 (i.q.r. 80–126) ml/min at the start of NMP, increasing significantly to 215 (170–276) ml/min at the end (*P* = 0.001). Median perfusate pH, lactate and sodium increased significantly, whereas glucose, potassium and calcium perfusate remained constant.

**Table 3:** Differences between perfusion parameters at start *versus* end of normothermic machine perfusion

	Start of NMP	End of NMP	P*
Flow (ml/min)	117 (80–126)	215 (170–276)	0.001
Perfusate pH	7.52 (7.05–7.58)	7.62 (7.53–7.84)	0.037
Perfusate lactate (mmol/l)	5.1 (4.5–5.6)	11.9 (10.3–13.4)	0.001
Perfusate glucose (mmol/l)	10.6 (5.9–13.0)	10.9 (5.8–12.8)	0.182
Perfusate potassium (mmol/l)	7.6 (7.2–9.7)	8.8 (7.0–9.9)	0.906
Perfusate sodium (mmol/l)	145 (143–147)	149 (146–153)	0.005
Perfusate calcium (mmol/l)	1.25 (1.16–1.29)	1.21 (1.15–1.28)	0.682

Values are median (i.q.r.). NMP, normothermic machine perfusion. * Wilcoxon signed rank test.

### Secondary outcome: graft outcome

#### Normothermic machine perfusion *versus* Eurotransplant Senior Program cohort

To select a larger reference control group, 53 consecutive patients in the ESP were selected, who had been transplanted between 1 January 2016 and 31 December 2018. *Table 2* presents baseline characteristics for this control group in comparison with the NMP group. Donor BMI was significantly lower in the NMP group than in the historical controls (median 22.2 (i.q.r. 18.1–26.1) *versus* 25.6 (23.6–28.2) kg/m^2^ respectively; *P* = 0.043). Other baseline characteristics were not significantly different. Despite an additional second cold ischaemia time in the NMP group, total cold ischaemia time was similar in the two groups (*P* = 0.893).

**Fig. 2:**
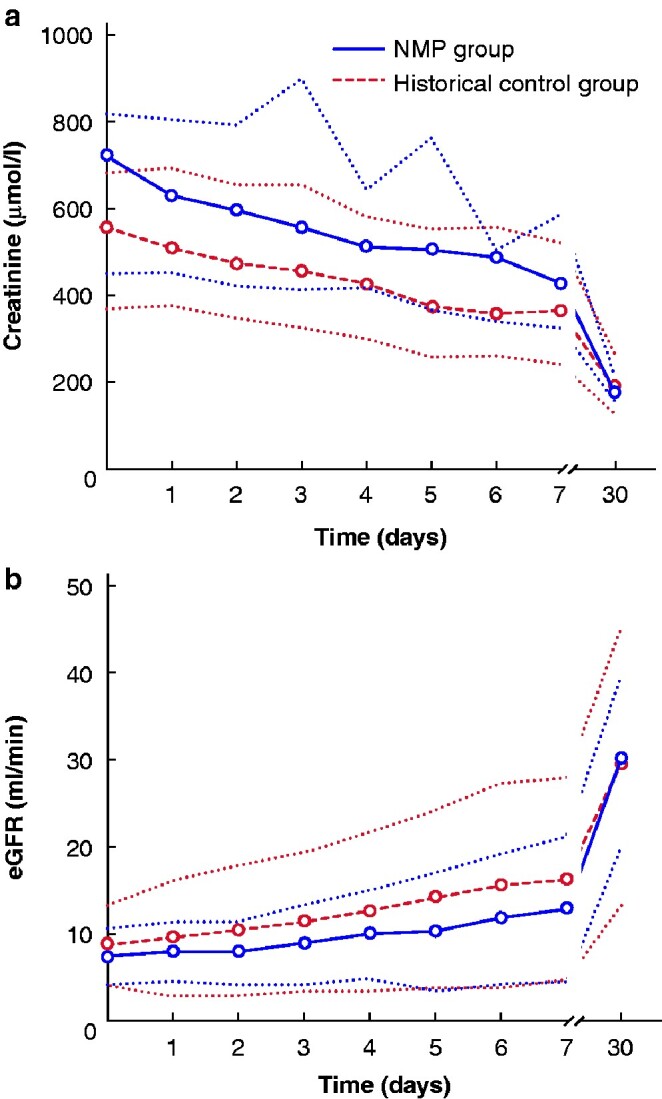
Comparison of creatinine levels and estimated glomerular filtration rate after transplantation in recipients of a NMP kidney and historical controls **a** Creatinine and **b** estimated glomerular filtration rate (eGFR). Median (i.q.r.) values are shown. eGFR, estimated glomerular filtration rate; NMP, normothermic machine perfusion.

**Fig. 3:**
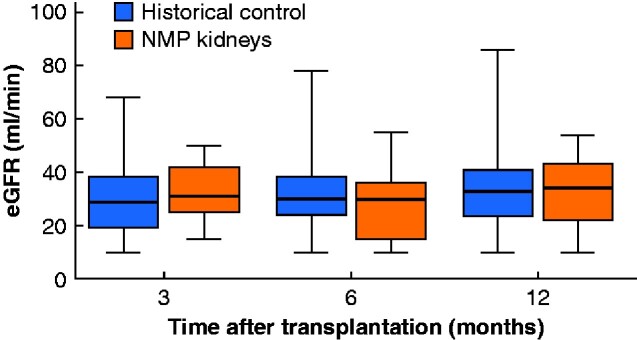
Box plots for eGFR at 3, 6 and 12 months after transplantation in recipients of a NMP kidney compared to historical controls Median values, interquartile ranges and ranges are denoted by horizontal bars, boxes and error bars respectively. eGFR, estimated glomerular filtration rate; NMP, normothermic machine perfusion.

Results of graft outcome in the NMP and ESP control groups are summarized in *Table 4*. Seven of the 11 patients (64 per cent) in the NMP group had immediate kidney function, compared with 21 of the 53 patients (40 per cent) in the historical ESP cohort (*P* = 0.144). No PNF occurred in the NMP group, compared with four cases in the historical controls (0 *versus* 8 per cent respectively; *P* = 0.347). The proportion of patients with DGF was 4 of 11 (36 per cent) in the NMP group *versus* 53 per cent in the historical controls (*P* = 0.320). Median duration of DGF was 15.5 (i.q.r. 8.3–98.5) days in the NMP group and 14.5 (5.5–26.8) days in the controls (*P* = 0.602). One of the four patients with DGF in the NMP group was pre-emptive and developed DGF as a result of atrial fibrillation with haemodynamic instability, requiring cardiopulmonary resuscitation followed by admission to the ICU. *Fig. 2* shows similar creatinine and eGFR trajectories in the first week after transplantation and at 30 days in NMP and historical control groups. Groups did not differ in patient or (death-censored) graft survival, incidence of BPAR, or proportion of patients with an eGFR above 30 ml/min after 3, 6 and 12 months (*Table 4*). No differences in eGFR were observed between the groups at 3 months (*P*=0.510), 6 months (*P*=0.482) and 1 year (*P*=0.908) (*Fig. 3*).

**Table 4:** Comparison of clinical outcomes in normothermic machine perfusion group and historical controls

	NMP group (*n* = 11)	Historical control group (*n* = 53)	*P* ^†^
Immediate function	7 (646)	21 (40)	0.144
PNF	0 (0)	4 (8)	0.347
DGF	4 (36)	28 (53)	0.320
Duration in DGF (days)^*^	15.5 (8.3–98.5)	14.5 (5.5–26.8)	0.602^‡^
eGFR ≥30 ml/min			
3 months	7 (64)	26 (49)	0.379
6 months	6 (55)	29 (55)	0.992
1 year	6 (55)	31 (58)	0.809
BPAR	4 (36)	14 (26)	0.504
1-year graft survival (%)	81	85	0.575^§^
1-year graft survival, DC (%)	91	91	0.537^§^
1-year patient survival (%)	91	91	0.560^§^

Values in parentheses are percentages unless indicated otherwise;

*values are median (i.q.r.). NMP, normothermic machine perfusion; PNF, primary non-function; DGF, delayed graft function; eGFR, estimated glomerular filtration rate; BPAR, biopsy-proven acute rejection; DC, death-censored.

^†^χ^2^ or Fisher’s exact test, except

^‡^Mann–Whitney *U* test and ^§^log rank test.

#### Normothermic machine perfusion *versus* contralateral kidneys

**Fig. 4:**
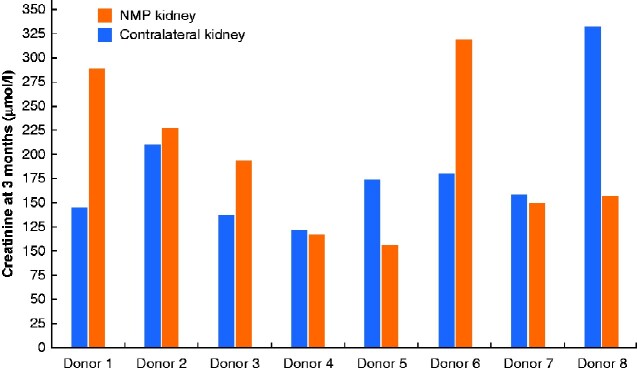
Creatinine level at 3 months in recipients of a NMP kidney compared to the contralateral kidney. NMP, normothermic machine perfusion.

Eight kidneys were eligible for comparison with the contralateral kidney, as one kidney was not transplanted (unknown reasons) and the other two contralateral kidneys both underwent NMP in this study. Second warm ischaemia time was significantly shorter in the NMP group (median 19 (i.q.r. 17–22) min *versus* 40 (37–50) min for the contralateral kidney; *P* = 0.002). Total cold ischaemia time was similar (median 597 (512–795) *versus* 679 (574–700) min respectively; *P* = 0.867). DGF occurred in two kidney pairs in both kidneys, and in one pair only in the NMP kidney; biopsy of this kidney showed chronic damage with acute tubular necrosis without rejection. The 1-year death-censored graft survival rate was 100 per cent in the NMP group and 80 per cent in the contralateral kidney group (*P* = 0.206, log rank test). The 1-year uncensored graft survival rate was 86 per cent in the NMP group and 80 per cent in the contralateral kidney group (*P* = 0.637). The 1-year patient survival rate was 86 per cent in the NMP group and 75 per cent in the contralateral kidney group (*P* = 0.731) Rejection occurred in two kidney pairs in both kidneys and in one pair only in the NMP kidney. At 3 months, creatinine levels were determined for all eight kidney pairs and found to be similar between two kidney pairs (*Fig. 4*). For four kidney pairs the creatinine concentration was lower in the NMP group, and for two kidney pairs creatinine levels were higher in the NMP group. The NMP kidney of donor 8 had a prolonged DGF based on acute tubular necrosis with discontinuation of haemodialysis after 3 months. Unfortunately, creatinine levels remained above 300 μmol/l. The NMP kidney of donor 5 experienced vascular rejection at the 3-month creatinine measurement; this recovered fully, with a creatinine concentration at 1 year of 120 μmol/l. Paired analysis showed a median 3-month creatinine level of 166.5 (i.q.r. 139.8–203.5) μmol/l in the NMP group and 176 (125.3–273.8) μmol/l in the contralateral kidney group (*P* = 0.779).

## Discussion

This pilot study described experience with 2 h of NMP and supports the feasibility and safety of implementation of NMP in this frail patient group. No adverse events were noted during or after NMP. Moreover, analyses for graft outcome showed overall comparable kidney function in NMP and standard preserved kidneys, indicating the safety of NMP in this ESP group. An interesting finding is the observed difference in immediate function between NMP kidneys and the historical controls. Although this did not reach statistical significance owing to the small sample size, the magnitude of the difference suggests that the technique of NMP may be clinically relevant.

The results concerning the impact of NMP on the incidence of DGF are comparable with the first clinical study of 18 ECD–DBD donor kidneys by the Cambridge group^11^, who found a statistically significant difference in DGF with an incidence of 36 per cent in the control group and 6 per cent in the NMP group (*P* = 0.014). The impact of NMP on DGF is probably mediated by interference with ischaemia–reperfusion injury (IRI). ESP kidneys are more vulnerable to IRI, due to increased donor age and co-morbidity of the donor. Even though no active therapy was given during NMP, a recent study^17^ showed that kidneys after NMP followed by simulated transplantation had upregulated pathways, promoting cell proliferation, compared with kidneys that were kept on SCS. This shows that NMP itself may already have a positive effect on the donor kidney, and may explain the difference in DGF incidence.

One of the strengths of this study is the use of a paired analysis with the contralateral kidney as a control group. The comparisons made with the historical cohort are confounded; this was addressed by also analysing the outcomes for the contralateral kidney of the same donor. Another strength is that cannulation and attaching the kidney to the Kidney Assist was carried out by one surgeon, which reduces variability in this technique that may have affected quality of NMP. A limitation may be that the donor kidneys were stored for a short period on SCS after NMP for logistical reasons. This could have counteracted the benefit of NMP. However, this risk is deemed small as previous studies^18–21^ have shown that NMP may be also beneficial if carried out at an earlier stage in the total cold ischaemia time.

Unfortunately, one protocol violation took place where one of the kidneys was from a donor nearly 64 years of age instead of 65 years or older. This may have affected graft outcomes, as the cut-off age for the ESP is 65 years. However, as the primary outcome was safety and feasibility, it was decided to include this patient in the analyses. Finally, a recent study^22^ showed that DGF appears not to correlate well with long-term graft survival outcomes in DCD kidney transplantation. However, a lower incidence of DGF may decrease healthcare costs by decreasing the need for dialysis treatment following transplant and shortening the length of hospital stay^22^^,^^23^. A longer follow-up is needed to study the impact of NMP on long-term graft outcomes.

The present study protocol differed slightly from that presented by the Cambridge group^11^. First, the present study performed 2 h of NMP, in contrast to the Cambridge group who performed 1 h. Kaths and co-workers^24^ showed in an animal study that longer periods of NMP (up to 16 h) may be superior to brief NMP. A reason for longer duration of NMP is to allow time for ‘therapy on the pump’ in the future. Safety of these longer perfusion durations has not yet been confirmed in clinical studies. Therefore, the present study protocol used a slightly longer NMP than usual, but still a duration that is unlikely to cause harmful effects. Second, antibiotics were added in the present study because of potential contamination of the kidney during NMP. Previous studies have shown that positive cultures in perfusate fluid are prevalent, causing infection in up to 10 per cent of the recipients^25^.

A few adjustments could be considered to optimize the NMP procedure in the future. First, assuming that NMP of longer duration is beneficial, the addition of albumin may be recommended to increase the osmotic pressure, preventing oedema^26^^,^^27^. Second, in the present protocol urine output was recirculated as a previous study^16^ showed this to be superior in maintaining homeostasis, rather than replacing urine production with Ringer’s lactate. However, if longer perfusion times are to be realized, control of waste products may be necessary and the addition of a dialysis membrane in combination with a haemoadsorption filter may be beneficial to clear waste products and proinflammatory cytokines^28^. Lastly, the risk of allosensitization can be addressed by using an acellular perfusion solution containing a haemoglobin-based oxygen carrier, which has been shown to be at least as effective as a blood-based perfusate for liver NMP^29^.

The findings of this pilot study support the performance of a larger study to explore further the potential efficacy of NMP to improve graft outcome. As a next step, the focus should be on exploring the safety of prolonged NMP and the possibility of administering therapy that specifically targets IRI. Currently, an RCT is being carried out in the UK, comparing NMP with SCS. This trial will help to identify biomarkers and perfusion cut-off values to define required length of perfusion. NMP not only has the potential to improve outcome in kidneys accepted according to current standards, but might also reduce discard rates, as Hosgood *et al*.^9^ showed recently in a small study of 10 kidneys declined for transplantation, where NMP led to 50 per cent of kidneys eventually being transplanted.

## Funding

Coolsingel Foundation (‘Stichting Coolsingel’, project number 557)

This study was funded by the Coolsingel Foundation (‘Stichting Coolsingel’). The funder was not involved in the research design, data collection, analysis, or manuscript preparation.

## Notes

Presented to the European Society for Organ Transplantation Congress in Copenhagen, Denmark (September 2019) and the Dutch Transplant Conference in Roermond, the Netherlands (March 2020)

## References

[1] Tonelli M , Wiebe N, Knoll G, Bello A, Browne S, Jadhav D et al Systematic review: kidney transplantation compared with dialysis in clinically relevant outcomes. Am J Transplant 2011;11:2093–2109 21883901 10.1111/j.1600-6143.2011.03686.x

[2] Batabyal P , Chapman JR, Wong G, Craig JC, Tong A. Clinical practice guidelines on wait-listing for kidney transplantation: consistent and equitable? Transplantation 2012;94:703–713 22948443 10.1097/TP.0b013e3182637078

[3] Port FK , Bragg-Gresham JL, Metzger RA, Dykstra DM, Gillespie BW, Young EW et al Donor characteristics associated with reduced graft survival: an approach to expanding the pool of kidney donors. Transplantation 2002;74:1281–1286 12451266 10.1097/00007890-200211150-00014

[4] Cohen B , Smits JM, Haase B, Persijn G, Vanrenterghem Y, Frei U. Expanding the donor pool to increase renal transplantation. Nephrol Dial Transplant 2005;20:34–41 15522904 10.1093/ndt/gfh506

[5] Frei U , Noeldeke J, Machold-Fabrizii V, Arbogast H, Margreiter R, Fricke L et al Prospective age-matching in elderly kidney transplant recipients—a 5-year analysis of the Eurotransplant Senior Program. Am J Transplant 2008;8:50–57 17973969 10.1111/j.1600-6143.2007.02014.x

[6] Peters-Sengers H , Berger SP, Heemskerk MB, Al Arashi D, Homan van der Heide JJ, Hemke AC et al Stretching the limits of renal transplantation in elderly recipients of grafts from elderly deceased donors. J Am Soc Nephrol 2017;28:621–631 27729570 10.1681/ASN.2015080879 PMC5280003

[7] Moers C , Smits JM, Maathuis MH, Treckmann J, van Gelder F, Napieralski BP et al Machine perfusion or cold storage in deceased-donor kidney transplantation. N Engl J Med 2009;360:7–19 19118301 10.1056/NEJMoa0802289

[8] Gallinat A , Moers C, Treckmann J, Smits JM, Leuvenink HG, Lefering R et al Machine perfusion *versus* cold storage for the preservation of kidneys from donors ≥65 years allocated in the Eurotransplant Senior Programme. Nephrol Dial Transplant 2012;27:4458–4463 22844103 10.1093/ndt/gfs321

[9] Hosgood SA , Thompson E, Moore T, Wilson CH, Nicholson ML. Normothermic machine perfusion for the assessment and transplantation of declined human kidneys from donation after circulatory death donors. Br J Surg 2018;105:388–394 29210064 10.1002/bjs.10733 PMC5887977

[10] Chandak P , Phillips BL, Uwechue R, Thompson E, Bates L, Ibrahim I et al Dissemination of a novel organ perfusion technique: ex vivo normothermic perfusion of deceased donor kidneys. Artif Organs 2019;43:E308–E319 31087667 10.1111/aor.13499

[11] Nicholson ML , Hosgood SA. Renal transplantation after *ex vivo* normothermic perfusion: the first clinical study. Am J Transplant 2013;13:1246–1252 23433047 10.1111/ajt.12179

[12] Gregorini M , Corradetti V, Pattonieri EF, Rocca C, Milanesi S, Peloso A et al Perfusion of isolated rat kidney with mesenchymal stromal cells/extracellular vesicles prevents ischaemic injury. J Cell Mol Med 2017;21:3381–3393 28639291 10.1111/jcmm.13249 PMC5706569

[13] Sierra-Parraga JM , Eijken M, Hunter J,, Moers C, Leuvenink H, Moller B et al Mesenchymal stromal cells as anti-inflammatory and regenerative mediators for donor kidneys during normothermic machine perfusion. Stem Cells Dev 2017;26:1162–1170 28557562 10.1089/scd.2017.0030

[14] Hosgood SA , Nicholson ML. First in man renal transplantation after *ex vivo* normothermic perfusion. Transplantation 2011;92:735–738 21841540 10.1097/TP.0b013e31822d4e04

[15] Minor T , von Horn C, Gallinat A, Kaths M, Kribben A, Treckmann J et al First-in-man controlled rewarming and normothermic perfusion with cell-free solution of a kidney prior to transplantation. Am J Transplant 2020;20:1192–1195 31599063 10.1111/ajt.15647

[16] Weissenbacher A , Lo Faro L, Boubriak O, Soares MF, Roberts IS, Hunter JP et al Twenty-four-hour normothermic perfusion of discarded human kidneys with urine recirculation. Am J Transplant 2019;19:178–192 29758129 10.1111/ajt.14932 PMC6491986

[17] Hameed AM , Lu DB, Patrick E, Xu B, Hu M, Chew YV et al Brief normothermic machine perfusion rejuvenates discarded human kidneys. Transplant Direct 2019;5:e502 31773055 10.1097/TXD.0000000000000944 PMC6831120

[18] Hosgood SA , Nicholson ML. The first clinical case of intermediate *ex vivo* normothermic perfusion in renal transplantation. Am J Transplant 2014;14:1690–1692 24816186 10.1111/ajt.12766

[19] Maessen JG , van der Vusse GJ, Vork M, Kootstra G. The beneficial effect of intermediate normothermic perfusion during cold storage of ischemically injured kidneys. A study of renal nucleotide homeostasis during hypothermia in the dog. Transplantation 1989;47:409–414 2646771 10.1097/00007890-198903000-00001

[20] Rijkmans BG , Buurman WA, Kootstra G. Six-day canine kidney preservation. Hypothermic perfusion combined with isolated blood perfusion. Transplantation 1984;37:130–134 6364495 10.1097/00007890-198402000-00003

[21] van der Wijk J , Slooff MJ, Rijkmans BG, Kootstra G. Successful 96- and 144-hour experimental kidney preservation: a combination of standard machine preservation and newly developed normothermic *ex vivo* perfusion. Cryobiology 1980;17:473–477 7002468 10.1016/0011-2240(80)90057-7

[22] de Kok MJ , McGuinness D, Shiels PG, de Vries DK, Nolthenius JBT, Wijermars LG et al The neglectable impact of delayed graft function on long-term graft survival in kidneys donated after circulatory death associates with superior organ resilience. Ann Surg 2019;270:877–883 31567503 10.1097/SLA.0000000000003515

[23] Incerti D , Summers N, Ton TGN, Boscoe A, Chandraker A, Stevens W. The lifetime health burden of delayed graft function in kidney transplant recipients in the United States. MDM Policy Pract 2018;3:2381468318781811 30288451 10.1177/2381468318781811 PMC6124921

[24] Kaths JM , Echeverri J, Linares I, Cen JY, Ganesh S, Hamar M et al Normothermic *ex vivo* kidney perfusion following static cold storage-brief, intermediate, or prolonged perfusion for optimal renal graft reconditioning? Am J Transplant 2017;17:2580–2590 28375588 10.1111/ajt.14294

[25] Oriol I , Sabe N, Tebe C, Veroux M, Boin I, Carratala J. Clinical impact of culture-positive preservation fluid on solid organ transplantation: a systematic review and meta-analysis. Transplant Rev (Orlando) 2018;32:85–91 29275111 10.1016/j.trre.2017.11.003

[26] Schurek HJ , Alt JM. Effect of albumin on the function of perfused rat kidney. Am J Physiol 1981;240:F569–F576 7246741 10.1152/ajprenal.1981.240.6.F569

[27] Urcuyo D , Blum MF, Liu Q, Nassar A, Buccini LD, Diago Uso T et al Development of a prolonged warm *ex vivo* perfusion model for kidneys donated after cardiac death. Int J Artif Organs 2017;40:265–271 28574105 10.5301/ijao.5000586

[28] Hosgood SA , Moore T, Kleverlaan T, Adams T, Nicholson ML. Haemoadsorption reduces the inflammatory response and improves blood flow during *ex vivo* renal perfusion in an experimental model. J Transl Med 2017;15:216 29070045 10.1186/s12967-017-1314-5 PMC5657103

[29] Matton APM , Burlage LC, van Rijn R, de Vries Y, Karangwa SA, Nijsten MW et al Normothermic machine perfusion of donor livers without the need for human blood products. Liver Transpl 2018;24:528–538 29281862 10.1002/lt.25005 PMC5900573

